# An investigator-blinded, randomized study to compare the efficacy of combined CBT for alcohol use disorders and social anxiety disorder versus CBT focused on alcohol alone in adults with comorbid disorders: the Combined Alcohol Social Phobia (CASP) trial protocol

**DOI:** 10.1186/1471-244X-13-199

**Published:** 2013-07-30

**Authors:** Andrew J Baillie, Claudia Sannibale, Lexine A Stapinski, Maree Teesson, Ronald M Rapee, Paul S Haber

**Affiliations:** 1 Centre for Emotional Health, Psychology Department, Macquarie University, Sydney, Australia; 2 National Drug and Alcohol Research Centre, University of New South Wales, Sydney, Australia; 3 Drug Health Services, Royal Prince Alfred Hospital, Camperdown, Australia; 4 Sydney Medical School, University of Sydney, Camperdown, Australia

**Keywords:** Alcohol use disorders, Social anxiety disorder, Comorbidity, Cognitive behavior therapy, Clinical trial

## Abstract

**Background:**

Alcohol use disorders and social anxiety disorder are common and disabling conditions that frequently co-exist. Although there are efficacious treatments for each disorder, only two randomized controlled trials of interventions for these combined problems have been published. We developed a new integrated treatment for comorbid Social Anxiety Disorder and Alcohol Use Disorder based on established Motivational Interviewing (MI) and Cognitive Behaviour Therapy (CBT) interventions for the separate disorders. Compared to established MI/CBT for alcohol use disorders this new intervention is hypothesised to lead to greater reductions in symptoms of social anxiety and alcohol use disorder and to produce greater improvements in quality of life. Higher levels of alcohol dependence will result in relatively poorer outcomes for the new integrated treatment.

**Methods/design:**

A randomised controlled trial comparing 9 sessions of individual integrated treatment for alcohol and social phobia with 9 sessions of treatment for alcohol use problems alone is proposed. Randomisation will be stratified for stable antidepressant use. Post treatment clinical assessments of alcohol consumption and diagnostic status at 3 and 6 month follow-up will be blind to allocation.

**Discussion:**

The proposed trial addresses a serious gap in treatment evidence and could potentially define the appropriate treatment for a large proportion of adults affected by these problems.

**Trial registration:**

Australian New Zealand Clinical Trials Registry: ACTRN12608000228381.

## Background

Comorbid alcohol use disorders and social phobia (or social anxiety disorder) are common and disabling conditions. Although there are efficacious treatments for each disorder, little is known about the best way to treat these disorders when they co-exist. Genetic epidemiology [1], and basic cognitive and behavioural science [2,3] suggest these two disorders interact rather than simply co-occur. There are currently two randomized controlled trials of interventions for these combined disorders [4,5] showing that focusing on alcohol alone produces superior outcomes than a focus on both disorders simultaneously. However, neither study integrated evidence based treatments for the separate disorders. Randall et al. [4] used a social phobia treatment program of unknown effectiveness with severe “alcoholics”, while Schadé et al. [5] tested an intervention targeting alcohol and phobias that we will argue was poorly integrated. The proposed study will test a new integrated treatment for comorbid Social Anxiety and Alcohol Use Disorders based on established Motivational Interviewing (MI) and Cognitive Behaviour Therapy (CBT) interventions for the separate disorders in a randomized controlled trial. Its findings have the potential to provide valuable information to service providers regarding the treatment needs of people with these combined problems.

Epidemiological research has shown that alcohol use disorders and social phobia are prevalent mental health problems in the community, affecting one in four and one in seven people respectively in their lifetime [6]. We have also reported the significant lifetime and concurrent comorbidity of these disorders [6-8]. In population samples, between one-fifth and one-third of people with an alcohol use disorder have social phobia in their lifetime, and over one-third of those with social phobia have alcohol use disorders in their lifetime [1,9-11]. These studies have typically found a closer association with alcohol dependence than abuse and an age at onset of social phobia that predates that of alcohol use disorders [1,12].

Teesson and colleagues reported 12-month prevalence rates for mental health comorbidity among the 10,641 Australian adults in the National Survey of Mental Health and Wellbeing [6,8,13]. They found that 3.7% of people with an alcohol use disorder also had social phobia, and 16% of those with social phobia had an alcohol use disorder. The odds ratio of the association between the two disorders was 3.2 (95% confidence interval 1.8-5.8) [8]. Respondents with alcohol use disorders and comorbid mental health problems were significantly more disabled and more likely to use health services than respondents with an alcohol use disorder and no comorbidity [8].

Comorbid social anxiety and alcohol use disorder are more common and disabling in clinical than population samples [14-17]. Although the nature of the relationship between anxiety disorders and alcohol use problems has been the object of some debate, recent evidence suggests consistently that social phobia is primary and substance-independent and has an age at onset in early adolescence, typically before the onset of alcohol use problems [1,18-21].

Despite several comprehensive reviews of the relevant literature, explanations for this relationship between alcohol use problems and anxiety disorders remains unclear. Broadly theories can be grouped into those that propose alcohol to be primary, others that see anxiety as primary, and those that see the two disorders as interacting. As it is clear that the acute effects of alcohol consumption and alcohol withdrawal can increase anxiety levels, some have argued that alcohol anxiety comorbidity is illusory [22,23]. Such an argument may apply more to anxiety symptoms that would otherwise be diagnosed as panic disorder or generalised anxiety disorder because of alcohol’s effect on arousal. As avoidance and other phobic behaviour in both social phobia and agoraphobia is related to but not determined by arousal it is harder to explain away these phobias as a direct effect of alcohol.

The tension reduction hypothesis [24] and the self medication hypothesis [25] both propose that alcohol is consumed because of its sedative effects. Many of the simpler theoretical models developed to explain the link between anxiety symptoms and alcohol use disorders (e.g., Tension Reduction, Stress Response Dampening, and Self-Medication) fail to consider that alcohol is a drug of addiction so that once developed alcohol problems may be maintained by processes other than anxiety. Thus alcohol problems may persist beyond treatment of the anxiety disorder [2]. Once comorbidity is established, alcohol use problems act to maintain or worsen anxiety disorders. Alcohol, specifically alcohol withdrawal, may directly induce symptoms of anxiety, while intoxication in the face of phobic stimuli may inhibit habituation of conditioned fear responses or disconfirmation of inflated threat expectancies that are thought to underlie anxiety disorders. Thus, while many expect alcohol will calm their anxiety, it probably acts to maintain anxiety disorders [26]. For these reasons, Kushner et al. [3] postulated that short-term anxiety reduction from alcohol use, together with long-term anxiety induction from chronic drinking and withdrawal, can initiate a vicious cycle of increasing anxiety symptoms and alcohol use that sustains these comorbid disorders. One hypothesis that emerges from this model is that to be effective, treatment would need to conceptualise the interaction between anxiety and drinking and treat the cognitions and behaviour relating to the both disorders in an integrated manner. The posposed trial will be the first to do this.

There are brief interventions [27,28] available for individuals with alcohol use problems that are supported by empirical evidence and have demonstrated cost-effectiveness [29]. Whether these interventions result in clinically significant improvement in individuals with comorbid problems remains to be determined [30]. There are few studies that have addressed the impact of comorbid anxiety disorders on alcohol treatment outcome [31-37]. Overall, their results suggest that comorbidity is associated with worse alcohol treatment outcome, relapse and readmissions [36,37]. Driessen and colleagues [31] examined the association between drinking behaviour and the course of anxiety and depression in 100 alcohol dependent patients with and without these comorbid disorders during the early and late post-detoxification periods. At six months post-treatment abstinence rates differed significantly between groups (60.5% non-comorbid vs 30.5% comorbid anxiety and depression vs 23.5% anxiety alone comorbidity). Although not definitive, it appears that comorbid anxiety disorders may be associated with a poorer treatment outcome for alcohol dependence.

There are also efficacious empirically supported CBT-based treatments for social phobia [38-43]. However, because anxiety treatment trials usually exclude individuals with substance use problems the efficacy of the anxiety treatment cannot be assumed to generalise to individuals with comorbid substance use disorders. To our knowledge, only one study to date has specifically examined the impact of a co-morbid alcohol use disorder on social phobia treatment outcomes. McEvoy and Shand [44] showed that severity of pre-treatment alcohol misuse independently predicted change in social interaction anxiety, but not performance anxiety.

Two randomised controlled trials have explored the efficacy of treatment for alcohol use problems and co-existing social phobia [4,5]. In the first RCT of alcohol use disorders and social phobia [4], individuals meeting DSM-III-R criteria for these disorders were recruited from among outpatients seeking treatment for alcohol use problems. Participants were randomised to one of two treatment conditions: 12 weeks of individual CBT treatment for alcohol dependence or 12 weeks of treatment for both alcohol and social phobia. Data were collected at the end of treatment and 3 months later. Participants in both treatment conditions improved significantly on social phobia and alcohol measures. However, the concurrent treatment group had a significantly poorer outcome on some alcohol measures compared to the alcohol only group: participants drank more frequently, had more heavy drinking days, and drank more drinks than participants who received alcohol treatment only. Although there were no significant differences in rates of attendance between the two groups, more participants completed all 12 dual treatment sessions than alcohol only sessions (43% versus 32%).

The unexpected finding of worse alcohol outcome in the dual treatment group in Randall et al. [4] was attributed to several limitations, including: parallel presentation of two unintegrated manualized treatments, in which alcohol was addressed during the first 45 minutes of each session and social phobia in the next 45 minutes; and a loss of alcohol treatment time in the dual treatment condition. The authors comment that the sample was drawn from a “severe population of treatment seeking alcoholics” (p 218). It is possible those with more severe alcohol dependence may not do as well if they are distracted from a focus on their treatment for alcohol dependence. In addition the intervention for social phobia was based on an unpublished treatment manual that had not previously been evaluated. This intervention may have constituted suboptimal social phobia treatment and an intervention supported by empirical evidence may have yielded different results. The findings of worse alcohol outcomes following dual treatment are surprising and merit replication.

The second study of alcohol and phobias [5] was a randomised controlled trial of 96 abstinent alcohol dependent patients with comorbid social phobia or agoraphobia. The patients were randomly assigned to an intensive, comprehensive 32 week psychosocial program, involving 3–4 months of inpatient groups followed by up to 16 weeks of aftercare, relapse-prevention and disulfiram on its own or in combination with an anxiety treatment program comprising CBT and optional pharmacotherapy. The anxiety treatment consisted of 12 weekly 60-minute sessions of cognitive therapy delivered individually at an anxiety clinic; the first six sessions of treatment dealt with alcohol only. The authors found that additional therapy for anxiety significantly reduced anxiety symptoms and avoidance behaviour but did not affect the alcohol relapse rates. While 13 of the 49 (26.5%) who were allocated to Alcohol only treatment were abstinent for 30 days before each of the three assessments, 18 of the 47 (38.3%) who were allocated to alcohol and anxiety treatment were similarly abstinent giving an effect size of 0.13. While this difference was not statistically significant, there was only a 23.4% chance that this size of effect would be detected with the sample size employed. In addition there was a trend of greater reduction of heavy drinking days in the dual treatment group: the reduction in mean number of days drinking 5 or more drinks from baseline to follow-up, was greater (not significantly) in the combined (mean reduction 9.4) than in the alcohol only treatment (mean 7.1). The inclusion of both panic/agoraphobia and social phobia comorbid with alcohol leaves the results less clear. The genetic epidemiology [1] suggests that while alcohol use disorders and panic disorder may share vulnerability, social anxiety and drinking have distinct vulnerabilities. Thus, the processes that maintain comorbid disorders may be different and thus require different treatment. It may also be that patients who require eight months of treatment for alcohol may require more than the six sessions of cognitive therapy for social phobia provided in this trial and, conversely, that those who have less chronic or severe alcohol problems may derive greater benefits from dual treatment than did patients in the above study.

These studies suggest that treating anxiety disorders in alcohol dependent patients undergoing alcohol treatment improves the anxiety disorder but may not yield superior alcohol treatment outcomes relative to alcohol treatment alone. The results of the above two trials are not definitive. The first study [4] employed a previously unevaluated intervention for social phobia and delivered treatment in a parallel and unintegrated manner to a sample of severe treatment seeking “alcoholics”. In the second study [5] anxiety treatment was delivered at a different clinic by different clinicians to patients with both social anxiety and panic/agoraphobia. Thus, these studies may not constitute optimal tests of integrated, empirically based interventions for these prevalent problems.

If ongoing anxiety problems have a deleterious impact on the maintenance of alcohol treatment effects then it is important that efficacious treatment be identified. The present study aims to test the efficacy of an empirically supported intervention for social phobia integrated with an existing well-established CBT intervention for alcohol use problems supported by empirical evidence. The treatment will be tested in a sample of participants with a range of severity of alcohol problems from consumption at harmful levels to alcohol dependence.

Cognitive behaviour therapy has been demonstrated to be an effective treatment for Social Phobia [45-51]. Some of the effective CBT interventions are based on Rapee and Heimberg’s [50] cognitive model for the maintenance of social phobia. The model proposes that individuals with social phobia experience distortions and biases in the processing of social or evaluative information which lead to increased anxiety and help to maintain social phobia. According to the model, treatment involves redirecting attentional resources away from the mental representation of how the individual appears to the audience and indicators of negative evaluation from the audience and towards the task at hand and more positive aspects of the audience. The model emphasises the importance of cognitive restructuring and objective feedback of performance as well as instruction and feedback regarding avoidance behaviours when exposure is undertaken. Alcohol use, in Rapee and Heimberg’s [50] model, is construed as an avoidance behaviour or a means of self handicapping [52]. A person with social phobia would learn to use alcohol as a safety behaviour and avoidance strategy in social or evaluative situations in an attempt to make the situation easier to cope with. Alternatively, Alcohol use would allow a face saving excuse for their perceived or actual poor social performance (self-handicapping). The acute effects of alcohol in a social situation could also reduce attentional resources so that corrective information about actual performance or the responses of others is ignored. With continued use of alcohol, anxiety and depression would increase and further reinforce beliefs that they are incompetent and poorly skilled [50]. This package of treatment is of known efficacy and is likely to be more effective than the previously unproven intervention used in the Randall et al. [4] trial.

There are also effective pharmacological [53,54] and psychological treatments for alcohol use disorders. Our own research [53], and that reported in recent a large US trial [54] has found naltrexone to be effective in reducing drinking in some with alcohol dependence. Naltrexone is a non-specific opioid antagonist with high affinity for the mu-opioid receptor, which is hypothesised to be the principal site of action. Chronic alcohol abuse has been linked to increased endogenous opioids, and naltrexone has been found to reduce the craving [55] and the ‘high’ produced by alcohol in humans [56]. The majority of controlled clinical trials have shown a significant advantage of naltrexone over placebo in the prevention of relapse to heavy drinking [53,54,57]. While other studies have not shown benefit over placebo [58,59] we believe there is sufficient evidence to offer all participants a trial of naltrexone (50 mg, 1 tablet daily) unless contraindications are present [60,61].

Baillie and Sannibale [62] have developed integrated cognitive behavioural treatment for comorbid alcohol and anxiety disorders and we further review the research and clinical rationale for the specific intervention strategies in Stapinski et al. [63]. This paper documents the protocol for evaluating the combined integrated MI/CBT for comorbid social anxiety and alcohol use disorders.

## Methods/Design

The proposed study is a randomised controlled trial which aims to determine the relative efficacy of an integrated intervention, BT for social phobia and co-existing alcohol use problems (Combined Alcohol Social Phobia; CASP) compared with CBT for alcohol use problem alone (Alcohol Alone). The study was funded by a National Health and Medical Research Council Project Grant (#488508) to the authors and was reviewed and approved by Macquarie University Ethics Review Committee (Human Research) HE28MAR2008-R05758 and by Sydney South Western Area Health Service, Ethics Review Committee (RPA Zone) X09-0331 & HREC/09/RPAH/553.

### Objective

The proposed study aims to determine the relative efficacy of an integrated individual cognitive behaviour therapy (CBT) for alcohol use problems and social phobia compared with individual CBT targeting alcohol use problems alone for people with social phobia and alcohol problems.

### Hypotheses

1. Participants who receive treatment for alcohol use problems alone will show reductions in alcohol consumption and symptoms of social phobia and improvements on secondary outcomes.

2. Participants who receive the integrated treatment for alcohol use problems and social phobia will show significantly greater improvements in terms of alcohol consumption, social phobia and quality of life compared with those in the alcohol treatment alone. They will maintain a lower rate of alcohol consumption for longer than the alcohol treatment alone group and will have greater improvements on measures of general health and functioning.

3. Participants in the integrated treatment condition will have a lower rate of treatment attrition than participants in the alcohol treatment alone intervention.

4. Higher levels of alcohol dependence will result in relatively poorer outcomes for the integrated treatment.

### Trial design

This is a randomised controlled trial utilising individual level randomisation to either combined treatment for alcohol and social phobia or treatment for alcohol use problems alone. Randomisation will be stratified for baseline Antidepressant use.

### Sample

**Figure 1:**
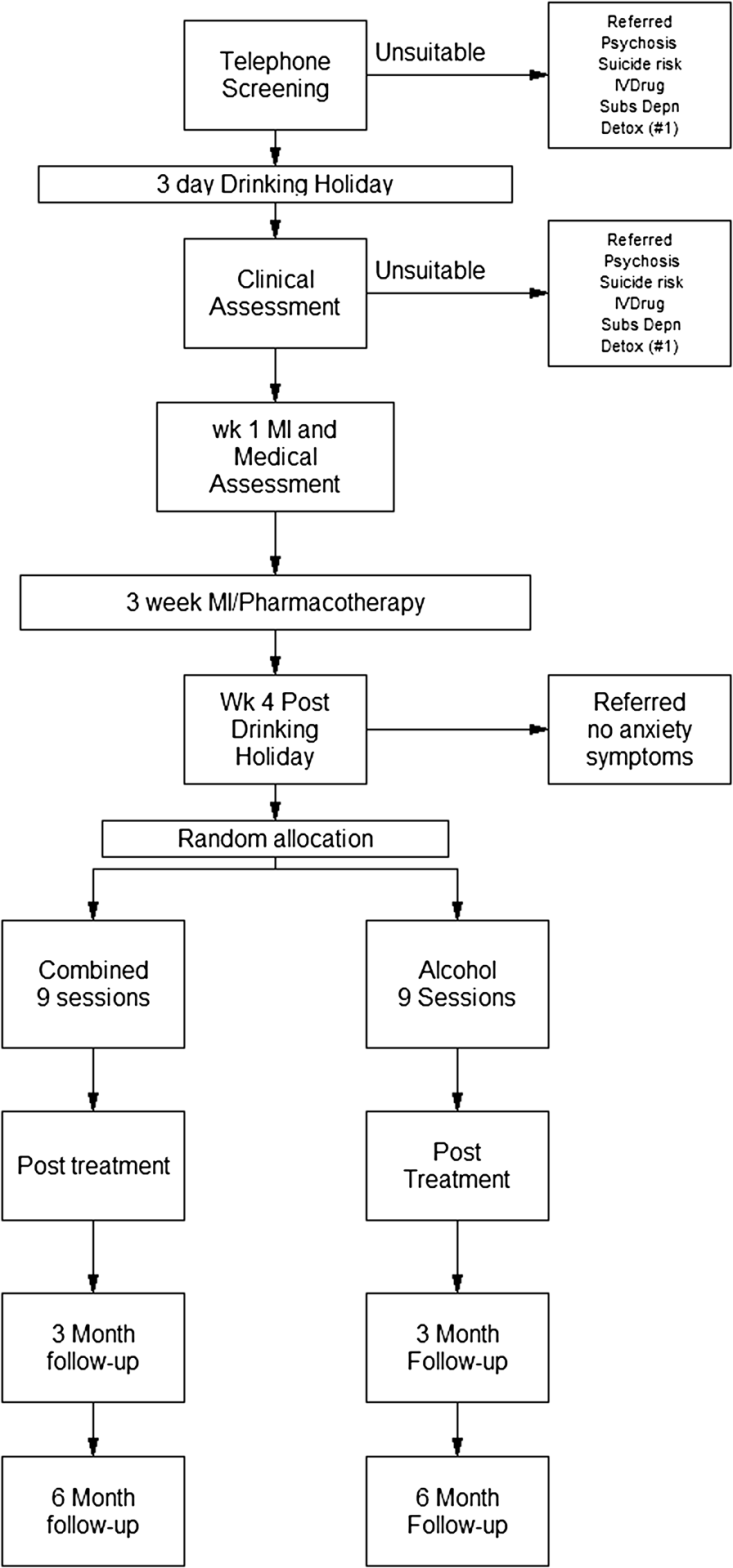
**Consort style flowchart.** Notes: #1 Potential participants who are excluded because of likely need for assistance in withdrawal are eligible to enter the study after successful withdrawal at the point of the clinical assessment.

To ensure that the full range of severity of alcohol problems and social phobia are included in the sample, recruitment will include screening in a range of health services (General Practice and specialist drug and alcohol and anxiety clinics) and advertising in media. Co-ordination of the trial and a telephone point of contact will be at the Centre for Emotional Health, Macquarie University (AJB, LS & RR). A CONSORT style flow chart is shown in Figure 1.

### Recruitment

Specialist alcohol and social phobia treatment services will be asked to screen all new presentations with the Alcohol Use Disorders Identification Test [64] and the Mini-SPIN [65] and contact the research team when there is a positive screen on both tests. Advertisements and media coverage will point potential participants to online versions of these two questionnaires or direct telephone screening.

### Inclusion/exclusion criteria

Participants will be adults (aged 18 years and older) who consume alcohol at harmful levels [66] (men consuming 29 standard drinks of alcohol per week or more and women 14 standard drinks per week or more), have a current (DSM-IV) diagnosis of social anxiety disorder and, have a basic literacy and ability to communicate in English to ensure comprehension and ability to complete reading and written take-home tasks.

Exclusion criteria for the study are: i) Active or florid psychosis (assessed using the Psychosis Screening Questionnaire [67]) or clinician suspicion following Anxiety Disorders Interview Schedule for DSM-IV (ADIS-IV) [68]. ii) History of schizophrenia spectrum disorder or bipolar disorder. iii) Current active suicidal intent (also assessed by a Clinical Psychologist with the ADIS-IV Anxiety Disorders Interview Schedule for Clinical diagnosis and Assessment of suicidal ideation). iv) Current injecting drug use (assessed using the Opiate Treatment Index, OTI) [69] v) Dependence on benzodiazepines or other substances (other than tobacco) (OTI) vi) Need for intensive detoxification (a score ≥ 20 on CIWA-AR [70]) (upon completion of detoxification participants will be eligible for inclusion); and vii) Inability to provide locator information or inability to participate in treatment and booster sessions (eg impending likelihood of imprisonment or residential treatment).

Given the high use of SSRI and other antidepressant medication in this population, the study population will be stratified into those who are taking antidepressants and those who are not. Patients who report regular use of antidepressant medication for at least one month, will be asked to remain on a stable dose during treatment. Patients taking these medications irregularly and without clear benefit will be asked to discontinue for two weeks prior to assessment for this study in consultation with the prescribing doctor.

### Assessment

Prospective participants will first contact the investigators by telephone for initial screening and if likely to be suitable they will be asked to complete questionnaires online via the Survey Monkey website and attend for an initial clinical assessment at either the Emotional Health Clinic at Macquarie University (AJB, LS, RR) or Drug Health Services at Royal Prince Alfred Hospital (CS, PH). Participants who meet inclusion criteria will be asked to reduce their drinking to “low risk” levels as recommended by NHMRC [66] (males up to 4 standard drinks per day and women up to 2 standard drinks per day) for three days to minimise the transient effects of alcohol use on functioning (e.g., intoxication, anxiety, irritability, sadness and depressed mood) [23,71].

Participants who are likely to require withdrawal management (severe alcohol dependence, previous withdrawal, current score ≥ 20 on CIWA-AR indicating established withdrawal) [70] will be referred to local specialist drug and alcohol clinics or participating general medical practitioners (selected GPs who have been made aware of this study and are willing to provide treatment for study participants). Once withdrawal is completed and the prospective participant has achieved 3 days of no more than low risk drinking, they are invited for the initial clinical interview.

The initial clinical assessment will include the Anxiety Disorders Interview Schedule for DSM-IV (ADIS-IV) [68] and be conducted by a Masters or PhD level Clinical Psychologist. In previous research [51] we have demonstrated high inter-rater reliability for social anxiety disorder (kappa = 0.87) and anxiety disorders in general (kappa = 0.80) using this interview. Participants will also be assessed using questions based on the Avoidant Personality Disorder section of the International Personality Disorder Examination (IPDE) [72].

All interviews will be audio-taped and a random sample of 25% will be rated by a PhD level Clinical Psychologist with relevant diagnostic experience, to determine diagnostic reliability. Measures used in the study are shown in Table 1 and include: the Anxiety Sensitivity Index [73]; Kushner’s Alcohol Outcome Expectancies Questionnaire [74]; the Timeline Follow-Back (TLFB) method for quantifying alcohol consumption over the previous 28 days [75,76]; the Opiate Treatment Index (OTI) to assess drug (and medication) quantity and frequency during the previous 28 days [69]; the Penn Craving Scale [77]; Self efficacy for abstinence [78]; the University of Rhode Island Change Assessment [79], used to assess motivation; Health Service Utilisation questions [80], used to asses other treatment received in study period.

**Table 1:** Schedule of assessments

**Assessment**	**Telephone contact**	**Pre treatment**	**Post washout**	**Post treatment**	**3 month follow-up**	**6 month follow-up**
*Eligibility*						
Alcohol Use Disorders Identification Test (AUDIT) [64]	+	-	-	-	-	-
Mini Social Phobia Inventory (Mini-SPIN) [65]	+	-	-	-	-	-
Clinical Institute Withdrawal Assessment for Alcohol Revised (CIWA-AR) [70]	+	-	-	-	-	-
Suicide Behaviours Questionnaire-Revised (SBQ-R) [81]	+	-	-	-	-	-
Psychosis Screening Questionnaire [67]	+	-	-	-	-	-
Anxiety Disorders Interview Schedule for DSM-IV (ADIS-IV) [82] incorporating International Personality Disorders Examination Avoidant Personality Disorder questions (IPDE) [72]	-	+	-	-	+	-
*Primary outcome measures*						
Social Phobia Scale and Social Interaction Anxiety Scale (SPS/SIAS) [83]	-	+	+	+	+	+
Time Line Follow Back (TLFB) [76]	-	+	+	+	+	+
Severity of Alcohol Dependence Questionnaire (SADQc) [84]	-	+	+	+	+	+
General Health functioning SF12 [85].	-	+	-	+	+	+
Blood for LFTs, FBC, CDT, Electrolytes	-	+	-	-	+(1)	-
*Secondary outcome measures*						
Sheehan Disability Scale(SDS) [86]	-	+	-	+	+	+
Depression, Anxiety and Stress Scale (DASS-21) [87]	-	+	+	+	+	+
Days out of role from the Brief Disability Questionnaire [88]	-	+	-	+	+	+
Drinker Inventory of Consequences (DRINC) [89]	-	+	-	+	+	+
*Static and varying covariates*						
Family Tree Questionnaire (FTQ)		+				
Anxiety Reduction Subscale of the Alcohol Expectancies Questionnaire [90]	-	+	-	+	+	+
Kushner’s Alcohol Outcome Expectancies Questionnaire [74]	-	+	-	+	+	+
Penn Alcohol Craving Scale(PACS) [77]	-	+	-	+	+	+
Alcohol Abstinence Self Efficacy Scale (AASES) [78]	-	+	-	+	+	+
Alcohol Expectancies for Social Evaluative Situations scale (AESES) [91]	-	+	-	+	+	+
Anxiety Sensitivity Index (ASI) [73]	-	+	-	+	+	+
University of Rhode Island Change Assessment (URICA) [79]	-	+	-	+	+	+
*Potential confounds*						
Health Service Utilisation questions [80]	-	+	-	+	+	+
The Opiate Treatment Index (OTI) [69,92]	-	+	-	+	+	+

Notes (1) Blood tests were used to verify abstinence only if baseline tests were elevated when drinking was reported.

Blood will be taken for full blood count (FBC), liver function tests (LFTs) and carbohydrate-deficient transferrin (CDT; a biological marker of recent alcohol consumption), calcium and electrolytes at baseline. If abnormal test results are obtained at baseline when self reported alcohol consumption is at harmful or hazardous levels blood will also be taken at follow-up. If there are no abnormalities at baseline with self reported alcohol consumption at harmful or hazardous levels no blood will be taken at follow-up as repeat testing not clinically indicated and not informative as a measure of alcohol consumption.

### Primary outcome measures

The primary outcomes for social anxiety disorder will be reductions in measures of social anxiety disorder: the Social Phobia Scale and Social Interaction Anxiety scale [83]. The primary outcome measures for alcohol are a reduction in the number of standard drinks per drinking day, assessed using the TLFB [75,76] and in the Severity of Alcohol dependence Questionnaire (SADQc) [84]. Quality of Life will be assessed by the SF12 [85].

### Secondary outcome measures

Secondary outcome measures are: the number (proportion) of days abstinent during the past 90 days; two time to event measures: (i) time to first drink; (ii) time to first heavy drinking day “relapse”, defined as three consecutive days drinking ≥ 6 standard drinks for a man and ≥ 4 standard drinks in a day for women) assessed using the TLFB [75,76]; the Depression, Anxiety and Stress Scale scores [87], and Days out of role from the Brief Disability Questionnaire [88].

### Procedure

After baseline assessment, all participants will complete a pre-treatment session of motivational interviewing followed by a 1 month “washout” period of abstinence. Symptoms of social anxiety (SPS and SIAS), general anxiety and depression (DASS) will then be reassessed. After reading and signing a written information and consent form, participants will be individually randomised by the sealed envelope method to one of two treatment conditions, of either: i) Treatment for alcohol use disorder comprising 9 weekly sessions of individual CBT for alcohol use disorder; OR ii) Treatment for alcohol use disorder and social anxiety disorder comprising 9 weekly sessions of integrated individual CBT for alcohol use disorder and anxiety disorder.

Each therapy session will last 60 to 90 minutes and will be conducted by one therapist (Clinical Psychologist). Both treatment conditions will have the same therapists and equal exposure to face-to-face treatment, approximately 10–15 hours, over a total of approximately three to five months.

Participants will be encouraged to attend treatment sessions weekly where possible, but will be given a maximum time frame of 5 months from initial assessment in which to complete treatment. Post and follow up assessment time points will be calculated from the end of all treatment sessions (1 motivational interviewing session plus 9 treatment sessions).

Participants will be considered non-completers if they miss more than 2 treatment sessions, or if they fail to complete the course of treatment within five months from assessment. Therapists will make at least 3 attempts to reschedule missed appointments, followed by a handwritten note two weeks after the last phone contact to i) acknowledge client’s decision to leave treatment, ii) encourage return to treatment, and iii) provide information about how this can be achieved. Participants who nevertheless choose to discontinue treatment will be contacted for post and follow up assessments calculated as if treatment were completed 3 months from the initial assessment.

### Treatment and treatment sites

Treatment will be provided at the Emotional Health Clinic, Centre for Emotional Health, Macquarie University, or Drug Health Services at Royal Prince Alfred Hospital (Sydney South West Area Health Service) and comprise one pre-treatment session followed by 9 weekly sessions. Two additional supplementary sessions can be used at treating therapist discretion to deal with crises that may emerge. The therapeutic approach will integrate CBT skills with a motivational interviewing style. Participants will complete monitoring diaries daily (recording the number of drinks consumed and rating their mood, anxiety and craving for alcohol), read educational material and complete take home tasks. The treatment goal is short-term abstinence (≥3 months), with participants selecting individual goals within recommended drinking guidelines beyond that period.

After an initial “washout” period of abstinence (≥ 1 month), goals for alcohol reduction will be determined collaboratively. Thus, participants may select a goal of abstinence, or controlled use within recommended drinking guidelines. Offering a choice of treatment goals will facilitate the inclusion of participants with various levels of dependence and decrease the risk of early drop-out [93].

### Participant retention

Participant retention will be maximised through a range of methods. Participants will be asked to provide at least two alternative contacts (e.g., Family member, partner or professional with whom they have regular contact) in addition to their own phone number and address, and if applicable email address. Regular contact (including study information letters, birthday cards and appointment reminder messages) with participants prior to, during and after their treatment will be maintained up until the final assessment at 6 months post treatment. During this time alternatives for data collection may be employed where they are in line with study protocol (e.g., conducting interviews by phone or email, or conducting sessions at sites more convenient for participants – e.g., home visits). Participants will be reimbursed $25 for their time attending follow-up assessments.

### Pre-treatment session

The aim of the pre-treatment session of motivation enhancement therapy is to place an unequivocal focus on the importance of addressing drinking and commit to an initial “washout” period of abstinence (≥ 1 month). There is little evidence that without substantial changes to substance use patterns treatment for anxiety disorders can be successful. This session is also designed to engage the individual in the treatment process at this crucial stage. In this session, which is based on the work of Miller et al. [94,95], the goal is to provide personalised feedback of the assessment of alcohol-related difficulties, check participants’ progress and increase or consolidate their commitment to reduced drinking and treatment in general; the session will also orient the person to the treatment program and introduce daily monitoring. A consultation with a medical practitioner will be organised to assess suitability for pharmacotherapy with naltrexone (50 mg daily). Participants will be reimbursed for costs associated naltrexone should it be prescribed.

### Treatment for alcohol use problems only

The components of this widely used CBT intervention include [96,97]: orientation to treatment approach; assessing high risk situations; coping with cravings and urges; managing thoughts about alcohol and drinking; problem solving; drink refusal skills; planning for emergencies; seemingly irrelevant decisions; dealing with a lapse and relapse prevention.

### Combined integrated treatment for alcohol use problems and social anxiety disorder

Based on the work of Rapee [47,51], Monti et al. [97] and our own review of the literature [62], we have developed an integrated cognitive behavioural treatment for comorbid social phobia and alcohol use disorders [63]. The intervention is based on a unified model of the interplay between social anxiety disorder and drinking. Initial sessions provide information about drinking and social anxiety disorder and their interaction, encourage awareness of individual triggers for drinking and anxiety through reflection on diaries and begin graded exposure and behavioural experiments within session as well as teaching techniques for challenging unhelpful thoughts, refusing drinks, and managing anxiety and cravings. Focusing on successes achieved during sessions helps to maintain motivation. Subsequent sessions extend graded exposure to more advanced behavioural experiments to progressively build coping skills. Each session involves techniques to build self-efficacy and self-mastery over anxiety and drinking. Detailed participant and therapist manuals have been developed and will be available from the authors at the completion of the trial.

Clinical supervision of the treating therapists will be provided for 1 hour per day with the 1^st^ or 2^nd^ author in order to assure the quality and fidelity of the interventions. Both AJB & CS are experienced clinical psychologists with additional training and experience in clinical supervision.

### Safety net

Participants who show deterioration in functioning requiring residential or psychiatric treatment will be referred to the appropriate specialist treatment services (anxiety clinics, psychiatric services or alcohol and other drug services). They will remain in the study but their outcome will be designated as relapsed (for anxiety, alcohol or both as appropriate). Either the last point carried forward or multiple imputation will be used to estimate possible scores so as to include these participants in analyses.

### Treatment integrity

A random selection of 20% of sessions will be audio-taped and rated for compliance with the treatment manual by an independent clinician (PhD level Clinical Psychologist) blind to treatment condition. The Cognitive Therapy Scale [98] will be employed to assess therapist competence.

### Follow-up

At the end of treatment and at three and six months after treatment completion, participants will be reassessed by an experienced independent research assistant blind to treatment condition. Participants will be reassessed on the main outcome measures in addition to the Drinker Inventory of Consequences (DRINC) [89], Kushner’s Alcohol Outcome Expectancies Questionnaire [74], the OTI, the Penn Craving Scale [77], Alcohol Expectancies Social Anxiety (AESES) [91], University of Rhode Island Change Assessment (URICA) [79], and the Health Service Utilisation questions [80].

### Analyses

Random effects regression models will be used to compare the efficacy of the treatment conditions on outcome measures from an intention to treat perspective. Random effect regression models (also known as hierarchical linear or mixed models) have been used in clinical trials involving relapse, and are suited to repeated measures, missing observations, and longitudinal data [99]. Random effects regressions can also account for variance associated with the clustering found with group treatment and multiple treatment sites. Cox regression will be used to determine time from baseline to first drink, time to first heavy drink. Last-point carried forward or multiple imputation will be used to determine the scores of participants who drop-out and are not located at follow-up.

### Determination of sample size

A sample size of 168 will be required to identify a moderate effect size of 0.5 between treatment groups, in a regression model with power = 0.8, alpha = 0.05. An additional 32 participants will be included to allow for an attrition rate of 20% during follow up. The total sample size required is therefore 200. This attrition rate is based on our recent experience in an alcohol dependent population with a high prevalence of comorbidity [53].

## Discussion

Alcohol use disorders and social phobia are commonly comorbid despite epidemiological and basic science findings that the two disorders interact to produce greater disability and poorer treatment outcomes. Existing clinical trials [4,5] indicate that alcohol focused treatment will give the best result. These trials did not test integrated treatments based on research into the mechanisms that may underlie the comorbidity and probably treated samples with severe alcohol dependence. Thus this trial will examine an integrated intervention based on basic cognitive and behavioural research in a sample with a range of severity of alcohol use disorders. It is hypothesised that this novel integrated package of motivational interviewing and cognitive behavioural therapy will lead to better overall outcomes across drinking, social anxiety and quality of life. In addition those with more severe levels of alcohol dependence are hypothesised to achieve relatively less benefit from the novel intervention than intervention focused on alcohol alone. This paper describes the trial protocol; companion papers describe the research and clinical basis for the contents of the intervention [63] and the intervention itself is documented in detailed therapist and participant manuals.

The proposed study addresses how individuals with comorbid alcohol use disorders and social phobia are best treated. Through the development and testing of the proposed intervention, the study could potentially define appropriate treatment for adults affected by these problems.

## Abbreviations

CASP: Combined alcohol social phobia.

## Competing interests

The authors declare that they have no competing interests.

## Authors’ contributions

AJB is the chief investigator of the CASP trial, he is responsible for the conduct of the study and the clinical supervision and training of the therapists and he wrote the first draft of this paper. CS drafted an earlier version of the grant proposal and collaborated with AJB, MT, RR & PH to produce the successful proposal. CS also provided clinical supervision and therapist training. LS edited and revised the proposal and is responsible for the day to day running of the trial. All authors read and approved the final manuscript.

## Pre-publication history

The pre-publication history for this paper can be accessed here:

http://www.biomedcentral.com/1471-244X/13/199/prepub
